# Comment on clinical characteristics and outcomes of non-tuberculous mycobacterial pulmonary infections after hematopoietic stem cell transplantation: A retrospective cohort study

**DOI:** 10.1016/j.htct.2025.106090

**Published:** 2026-01-10

**Authors:** Marhaba Khan, Tahir Abdul Qadir

**Affiliations:** Jinnah Sindh Medical University, Karachi, Pakistan

To the editor:

We eagerly read the paper entitled “Clinical characteristics and outcomes of non-tuberculous mycobacterial pulmonary infections after hematopoietic stem cell transplantation: A retrospective cohort study” by Zahia Esber [1] with great interest. The authors assessed the clinical manifestations, diagnosis, and consequences of lung infections caused by nontuberculous mycobacteria (NTM) in patients who received hematopoietic stem cell transplantation (HSCT). The study offers evidence-based recommendations for improving NTM infection diagnosis, risk assessment, and management in a susceptible post-transplant population.

As the authors point out, one of the study's main limitations is the difficulty in distinguishing between colonization and actual NTM pulmonary infection. One third (35.3 %) of the cases were categorized as “possible” infections (positive cultures alone) despite the application of the American Thoracic Society (ATS) and the Infectious Diseases Society of America (IDSA) criteria, which recent systematic reviews indicate may not necessarily imply clinically severe disease needing treatment. For example, single positive test results frequently indicate colonization, but ≥2 positive cultures are linked to a 98 % chance of *Mycobacterium avium* complex (MAC) pulmonary illness. Also according to the study's findings, the majority of patients (76.5 %) had positive results even though they did not receive therapy [1, 2, 3]. But the authors could make their arguments stronger by specifically citing this kind of research to put their management choices in perspective. Also, the lack of rapidly growing mycobacteria (RGM) instances (such as *Mycobacterium* abscessus) restricts the generalizability to more aggressive NTM species, a situation that calls for additional multicenter research.

There is a lack of a control group of HSCT patients with no NTM infection, which is another limitation of this study. Without this comparison, it is challenging to identify the clinical characteristics, risk factors, or results that are directly related to NTM infection as opposed to HSCT or related issues such as immunosuppression or graft-versus-host disease (GvHD). This makes it difficult to draw conclusions about causality and restricts the capacity to pinpoint the main risk factors or results linked to NTM. Liu et al., in their cohort analysis, addressed this restriction by comparing HSCT patients with and without mycobacterial infections. This made it possible to more clearly identify independent risk factors and outcomes linked to NTM infection. Their research highlights the significance of including a control group that is not infected in order to improve the validity and generalizability of results [4].

The authors in this study noted that 86.3 % of patients with NTM infections were on steroids and immunosuppressive therapy for GvHD. However, they did not conduct a thorough statistical analysis to determine whether GvHD and its treatment independently increased the risk of NTM infections. For example, a retrospective cohort study by Upton et al. found that chronic GvHD and cytomegalovirus (CMV) viremia were significantly associated with a higher risk of NTM disease, with hazard ratios of 1.99 and 5.77, respectively. Although routine NTM screening may be warranted in high-risk subgroups, such as those with GvHD or CMV coinfection, its usefulness should be substantiated by quantitative analysis of independent risk [5].

According to von Elm et al. in the STROBE Statement [6], “authors should report efforts to address potential sources of bias, such as design strategies, statistical adjustments, or sensitivity analyses.” The current study lacks any such reporting, making it difficult to assess the robustness of its conclusions. The authors should explicitly identify and discuss potential sources of bias inherent in the retrospective design, such as selection and information bias. The inclusion of strategies to minimize bias—such as blinded image review or comparison to a control group—would strengthen the study’s credibility and align it with best practices in observational research.

In contrast, Kontoyiannis et al. effectively handled bias in a similar HSCT cohort study by using matched controls, stratification, and acknowledging the limitations of their retrospective design [7]. Their approach enhances internal validity and should serve as a methodological benchmark.

## Funding statement

This research did not receive any specific grant from funding agencies in the public, commercial, or not-for-profit sectors.

## Ethical consent

Not applicable.

## Author contributions

Both authors contributed to the conceptualization, writing, and critical review of the manuscript. Both authors reviewed and approved the final version of the letter. The data that support the findings of this study are available from the corresponding author upon reasonable request.

## Conflicts of interest

None.
